# Tryptophan catabolism increases in breast cancer patients compared to healthy controls without affecting the cancer outcome or response to chemotherapy

**DOI:** 10.1186/s12967-019-1984-2

**Published:** 2019-07-23

**Authors:** Concetta Elisa Onesti, François Boemer, Claire Josse, Stephane Leduc, Vincent Bours, Guy Jerusalem

**Affiliations:** 1 Medical Oncology Department, Centre Hospitalier Universitaire Sart-Tilman, Liège, Belgium; 2 Laboratory of Human Genetics, GIGA Research Institute, Liège, Belgium; 3 Department of Human Genetics, Centre Hospitalier Universitaire Sart-Tilman, Liège, Belgium; 4 Liège University, Liège, Belgium

**Keywords:** Indoleamine 2,3-dioxygenase, Kynurenine, Tryptophan, Kynurenine/tryptophan ratio, Immune system, Breast cancer, Circulating biomarkers

## Abstract

**Background:**

Indoleamine 2,3-dioxygenase catalyzes the conversion of tryptophan to kynurenine, an immunosuppressive metabolite involved in T regulatory cell differentiation. Indoleamine 2,3-dioxygenase is expressed in many cancer types, including breast cancer. Here, we analyze kynurenine and tryptophan and their ratio in breast cancer patients and healthy controls.

**Methods:**

Breast cancer patients and healthy controls were prospectively enrolled in our study. All subjects underwent blood sample withdrawal at diagnosis or on the day of screening mammography for the healthy controls. Plasmatic kynurenine and tryptophan were determined on a TQ5500 tandem mass spectrometer after chromatographic separation.

**Results:**

We enrolled 146 healthy controls and 202 women with stages I–III breast cancer of all subtypes. All patients underwent surgery, 126 underwent neoadjuvant chemotherapy with 43 showing a pathological complete response, and 43 underwent adjuvant chemotherapy. We observed significantly higher plasmatic kynurenine, tryptophan and their ratio for the healthy controls compared to patients with breast cancer. We observed a lower plasmatic tryptophan and a higher kynurenine/tryptophan ratio in hormone receptor-negative patients compared to hormone receptor-positive cancers. Lobular cancers showed a lower ratio than any other histologies. Advanced cancers were associated with a lower tryptophan level and higher grades with an increased kynurenine/tryptophan ratio. Pathological complete response was associated with higher kynurenine values. The plasmatic kynurenine, tryptophan and kynurenine/tryptophan ratios were not predictive of survival.

**Conclusions:**

The plasmatic kynurenine, tryptophan and kynurenine/tryptophan ratio could differentiate breast cancer patients from healthy controls. The Kyn/Trp ratio and Trp also showed different values according to hormone receptor status, TNM stage, T grade and histology. These results suggest a rapid metabolism in breast cancer, but no associations with outcome or sensitivity to chemotherapy were observed.

**Electronic supplementary material:**

The online version of this article (10.1186/s12967-019-1984-2) contains supplementary material, which is available to authorized users.

## Background

Tryptophan (Trp) catabolism is a known mechanism involved in immune system modulation and is widely studied in cancer. Trp is metabolized by indoleamine 2,3-dioxygenase (IDO), by its splice variant IDO2 and by tryptophan 2,3-dioxygenase (TDO) [1]. IDO2 expression is narrower than IDO, and it is inactivated in a large percentage of the population due to some common polymorphisms, namely, R248W, that reduces IDO activity in approximately 90% of individuals, and Y359, which completely abolishes its catalytic function [2]. TDO is expressed at high levels in the liver, where it regulates systemic Trp levels, and in the brain, where it is responsible for the production of neuroactive metabolites, such as kinurenic and quinolinic acid [3]. IDO seems to play a central role in maintaining immune tolerance. Previous studies showed that IDO-deficient mouse models are more susceptible to autoimmune disease, while TDO-deficient mice showed only behavioral alterations [3, 4]. IDO expression is induced in response to some inflammatory cytokines, such as interferon-γ (IFN-γ), acting as an endogenous mechanism able to control an excessive immune response [5]. IDO is expressed by several tumor types and is correlated with lower cytotoxic cells and higher T regulatory (Treg) cell infiltration at the tumor site, poorer outcome and resistance to treatment [6–16].

Trp depletion induces an arrest at the G1 phase of the cell cycle, blocking T-lymphocyte proliferation, and triggers a stress response mediated by the activation of the nonderepressible kinase GCN2 pathway, which is responsible for T-cell anergy and apoptosis [17]. When exposed to low Trp levels, dendritic cells (DC) acquire tolerogenic capacity. DC showed an upregulation of the immunoglobulin-like transcript 3 and 4 inhibitory receptors (ILT3 and ILT4) and a lower expression of costimulatory molecules, such as CD40 and CD80 [18]. The high expression of ILT3 and ILT4 leads to the differentiation of CD4+CD25+Foxp3+ Treg cells [18]. Moreover, Trp catabolites, i.e. kynurenine (Kyn) and its downstream metabolites, acting through the activation of the aryl hydrocarbon receptor (AHR), are also responsible for Treg differentiation and in inhibition of T lymphocytes and natural killer (NK) cells [19, 20].

IDO is expressed in several tumor types, including breast cancer. A retrospective analysis conducted on 203 breast cancer patients showed that none of the tumor samples were negative for IDO staining in immunohistochemistry [21]. Larger, node-positive and estrogen receptor-positive (ER+) tumors were associated with higher IDO expression, while greater vascularized tumors showed lower IDO staining [21]. A greater CD8+ and CD11b+ cell infiltration was observed in low-IDO tumors, while Treg infiltration was not associated with IDO expression in this report [21]. Another study reported a negative association between high stromal IDO and worse disease-free and metastasis-free survival in breast cancer [22].

The plasmatic Kyn/Trp ratio is commonly used as a surrogate indicator of IDO activity, even though it might be an imperfect reflection of the tumor Trp catabolism. Lyon and colleagues observed a higher plasmatic Kyn/Trp ratio in patients affected by breast cancer compared to healthy controls, but not a statistically significant difference in the plasmatic level of the two molecules [23]. Another more recent study showed significantly lower plasmatic Trp and Kyn levels in breast cancer patients than in healthy controls, mainly in estrogen receptor-negative (ER−) tumors and at an advanced tumor stage [24]. Tang and colleagues observed higher Kyn plasmatic levels in ER− compared to ER+ cancers [25]. Overall, the previously mentioned studies did not unequivocally identify an association between tumor characteristics and the plasmatic Kyn, Trp and Kyn/Trp ratio, in addition to not showing an association with outcome, despite what was observed in other diseases [14–16].

In the present study, we analyzed the plasmatic levels of Trp, Kyn and their ratio in a large cohort of stage I–III breast cancer patients undergoing surgery ± neoadjuvant/adjuvant treatment and in healthy controls. The aim of our study was to identify differences in Kyn, Trp and the Kyn/Trp ratios in plasma between healthy and cancerous women to analyze the association of these parameters with outcome, their distribution according to tumor characteristics and their correlation with some tumor and patient characteristics.

## Materials and methods

### Patients selection

This was an observational prospective study in which stage I–III breast cancer patients treated with surgery with or without adjuvant/neoadjuvant treatment, radiotherapy and hormonal therapy according to clinical practice at the University Hospital of Liège in Belgium between May 2011 and August 2017 were enrolled.

Healthy controls were enrolled between January and November 2014 at the University Hospital of Liège in Belgium before performing a screening mammography and were allocated to the control group after confirmation of the absence of breast disease.

For all breast cancer patients, the following data were collected: ER status, progesterone receptor (PgR) status, HER2 status, Ki67, TNM stage, tumor size, tumor grade, type of treatment, response to chemotherapy for patients who received neoadjuvant chemotherapy (NAC), disease-free survival (DFS) and breast cancer-specific survival (BCSS).

This study was conducted in accordance with the Declaration of Helsinki. The institutional ethics committee approved the protocols, and each patient or healthy control signed informed consent before inclusion in the trial.

### Sample collection and storage

Blood sample withdrawal into an EDTA tube was performed before receiving any treatment for breast cancer patients or before the day of the screening mammography for healthy controls. After two centrifugation steps for 10 min at 815*g* and for 10 min at 2500*g* at 4 °C, plasma was collected in aliquots and stored at − 80 °C without thawing cycles until the sample analysis.

### Tryptophan and kynurenine determination

The plasmatic samples were used to determine the Kyn and Trp concentrations. After the step of protein precipitation with salicylic acid, the Kyn and Trp concentrations were determined on a TQ5500 tandem mass spectrometer after chromatographic separation. The values were calculated by the software Analyst, comparing the spectrometer intensity with known concentrations of internal standards for Trp and Kyn. The final concentrations were expressed in μM/L. Each sample was tested in triplicate, and the mean value was used for the statistical analysis.

### Statistical analysis

Statistical analyses and graphs were performed using IBM SPSS Statistic v24, MedCalc v19 and Prism v5 GraphPad software.

The co-primary end-points of our study were to assess the different distributions of plasmatic Kyn, Trp and the Kyn/Trp ratio in breast cancer (BC) patients compared to healthy controls (CTRL).

The secondary end-points were to identify the different distribution of plasmatic Kyn, Trp and the Kyn/Trp ratio according to patient and tumor characteristics (age, ER status, PgR status, HER2 status, Ki67, TNM stage, T stage, N stage, tumor grade, histology, subtype, lymphovascular invasion, response to NAC and relapse in the whole cohort and according to the different BC subtypes), to test the correlation between plasmatic Kyn, Trp and the Kyn/Trp ratio with the continuous variables age, ER, PgR, Ki67 and size and to assess DFS and BCSS in patients with Kyn, Trp and the Kyn/Trp ratio low vs. high.

The Mann–Whitney U test and Kruskal–Wallis test were used to compare the distributions of circulating Kyn, Trp and their ratio according to the following variables: cancer patients vs. healthy controls; age at diagnosis higher or lower than the median value; ER status; PgR status; HER2 status; Ki67; TNM stage; T stage; N stage; tumor grade; histology; subtype; lymphovascular invasion; response to neoadjuvant chemotherapy (NAC) and relapse. A post hoc Bonferroni test was performed for comparison by pairs for variables with multiple categories. An alpha level of 0.05 was accepted for significant results.

Spearman test was performed to analyze the correlation between plasma Kyn and Trp and their ratio with the following continuous variables: age, ER%, PgR%, Ki67% and tumor size in mm. An alpha level of 0.05 was accepted for significant results.

The median values of circulating Kyn, Trp and the Kyn/Trp ratio for cancer patients were calculated and used as cut-offs to classify patients into two groups for each variable. Kaplan–Meier curves for DFS and BCSS were drafted, and the statistical significance was tested by means of a log-rank test, with an accepted alpha level of 0.05. The 5-year DFS and the 5-year BCSS were calculated from the survival tables. The Cox Regression Hazard model was used to calculate the HR for both DFS and BCSS.

## Results

In total, 348 subjects, 146 of whom were healthy controls (CTRL) and 202 of whom were stage I–III breast cancer (BC) patients, were enrolled in this study between May 2011 and August 2017 at the University Hospital of Liège in Belgium.

The median age for the whole cohort was 54.5 years (range 26–86). In particular, the median age in the group of 202 breast cancer patients was 56 years old (range 26–86), while the median age in the healthy controls group was 53 years old (range 40–74). The median follow-up was 60 months (range 14–96). The patients were classified as stage I in 20.8%, stage II in 55% and stage III in 24.3% of the cases. All the breast cancer subtypes were included in the study: Luminal A in 29.7%; Luminal B in 44.1%; HER2-enriched in 6.9% and triple-negative breast cancer (TNBC) in 19.3% of the cases. All patients underwent surgery and received neoadjuvant/adjuvant chemotherapy, trastuzumab, radiotherapy and hormonotherapy according to cancer characteristics and physician choice. All patient characteristics and treatment details are summarized in Table 1.

**Table 1:** Patient baseline characteristics

	Number of patients	% of patients
Median age: 56 years		
Range: 26–86 years		
Histology		
Ductal carcinoma	172	85.1
Lobular carcinoma	23	35.4
Other	7	3.5
Subtype		
Luminal A	60	29.7
Luminal B	89	44.1
HER2-enriched	14	6.9
TNBC	39	19.3
ER status		
Positive	145	71.8
Negative	57	28.2
PgR status		
Positive	131	64.9
Negative	71	35.1
HER2 status		
Positive	55	27.2
Negative	147	72.8
Ki67		
< 20%	74	36.6
≥ 20%	127	62.9
Unk	1	0.5
Stage		
I	42	20.8
II	111	55
III	49	24.3
T stage		
1	64	31.7
2	89	44
3	21	10.4
4	27	13.4
Unk	1	0.5
N stage		
0	101	50
1	82	40.6
2	10	5
3	6	3
Unk	3	1.4
Tumor grade		
G1	5	2.5
G2	100	49.5
G3	91	45
Unk	6	3
Lymphovascular invasion		
Yes	41	20.3
No	156	77.2
Unk	5	2.5
Neoadjuvant chemotherapy (NAC)		
Yes	126	72.4
No	76	37.6
Type of NAC (N = 126)		
EC → Taxane	94	74.6
FEC → Taxane	21	16.7
EC → CBDCA-Ptx	6	4.8
Other	5	4
Pathological complete response (N = 126)		
Yes	43	34.1
No	83	65.9
Adjuvant chemotherapy (AC)		
Yes	43	21.3
No	159	78.7
Type of AC (N = 43)		
EC → Taxane	11	25.6
FEC → Taxane	20	9.9
Capecitabine	5	11.6
Other	7	3.5
Trastuzumab		
Yes	53	26.2
No	149	73.8
Radiotherapy		
Yes	174	86.1
No	28	13.9
Hormonotherapy		
Yes	149	73.2
No	54	26.8
Type of hormonotherapy		
SERM	56	37.6
AI	79	53
SERM + AI	14	9.4
Relapse		
Yes	28	13.9
No	174	86.1

*TNBC* triple-negative breast cancer, *Unk* unknown, *NAC* neoadjuvant chemotherapy, *AC* adjuvant chemotherapy, *EC* epirubicin–cyclophosphamide, *FEC* 5-fluorouracil–epirubicin–cyclophosphamide, *CBDCA* carboplatin, *Ptx* paclitaxel, *N* number of patients, *SERM* selective estrogen receptor modulator, *AI* aromatase inhibitor

**Fig. 1:**
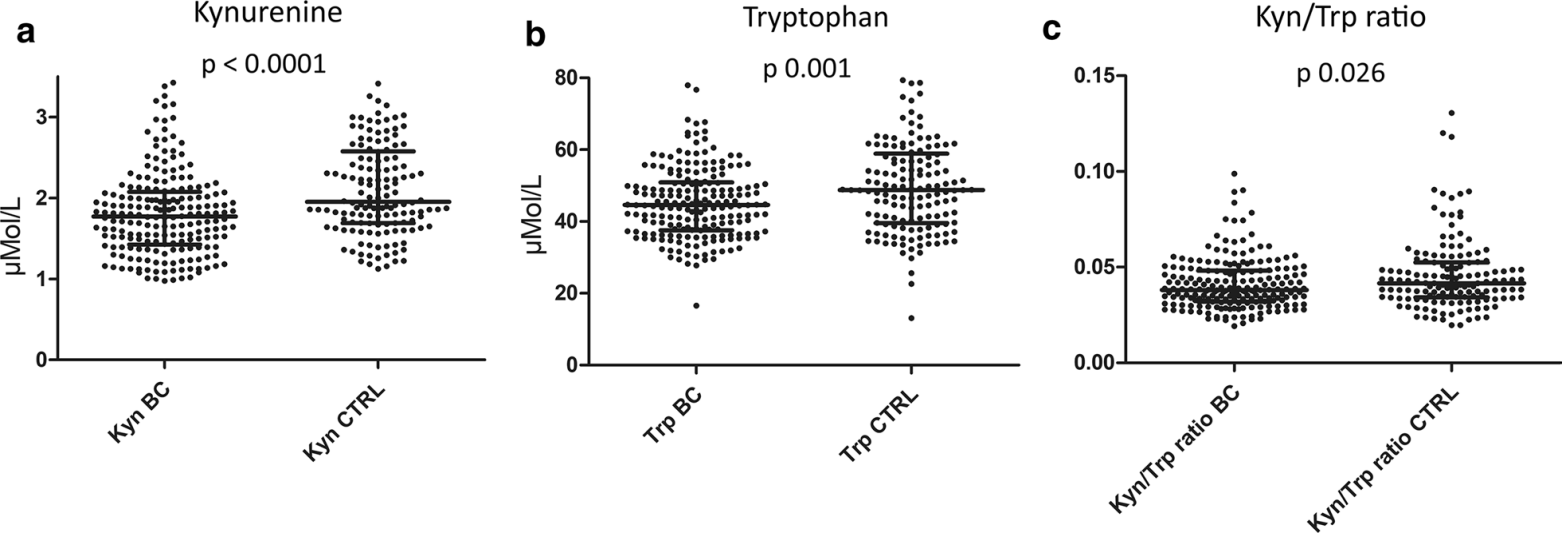
Scatter dot plots representing the differences in median value between breast cancer patients and healthy controls for Kyn (**a**), Trp (**b**) and the Kyn/Trp ratio (**c**). The values were compared by means of the Mann–Whitney U test, and the p value is reported in the figure

Circulating Kyn was significantly lower in the BC patients than in the healthy CTRLs, with a median value of 1.770 ± 0.537 μM/L vs. 1.951 ± 0.708 μM/L (p < 0.0001), respectively. The same observation was found for the Trp value in plasma, which was 44.56 ± 10.83 μM/L and 48.80 ± 14.16 μM/L in the BCs and CTRLs respectively (p 0.001), and for the plasmatic Kyn/Trp ratio, which was 0.038 ± 0.013 for the BC patients and 0.042 ± 0.019 for the healthy CTRLs (p 0.026) (Table 2 and Fig. 1).

**Table 2:** Kyn, Trp and Kyn/Trp ratio according to patient and tumor characteristics

Comparison group	Kyn (μM/L)		Trp (μM/L)		Kyn/Trp ratio	
	Median ± SD	P value	Median ± SD	P value	Median ± SD	P value
Healthy vs. cancer						
Healthy	1.951 ± 0.708	*<* 0.0001***	48.80 ± 14.16	0.001**	0.042 ± 0.019	0.026*
Cancer	1.770 ± 0.537		44.56 ± 10.83		0.038 ± 0.013	
Age (years)						
< 54.5	1.738 ± 0.609	*<* 0.0001***	45.68 ± 11.23	0.978	0.036 ± 0.015	< 0.0001***
≥ 54.5	1.980 ± 0.643		46.63 ± 13.78		0.043 ± 0.016	
Histology						
Ductal	1.810 ± 0.550	0.077	44.51 ± 10.65	0.051	0.038 ± 0.014	0.005**
Lobular	1.550 ± 0.451		48.60 ± 11.54		0.033 ± 0.009	
Other	1.543 ± 0.289		38.00 ± 10.92		0.038 ± 0.008	
Subtype						
Luminal A	1.770 ± 0.570	0.324	47.73 ± 12.48	0.213	0.038 ± 0.014	0.100
Luminal B	1.800 ± 0.458		44.57 ± 10.27		0.038 ± 0.011	
HER2-enriched	1.853 ± 0.881		40.42 ± 6.85		0.047 ± 0.023	
TNBC	1.707 ± 0.442		44.13 ± 9.90		0.038 ± 0.012	
Estrogen receptor expression						
ER-positive	1.800 ± 0.510	0.897	45.47 ± 11.26	0.016*	0.0377 ± 0.012	0.040*
ER-negative	1.757 ± 0.606		42.00 ± 8.88		0.042 ± 0.016	
Progesterone receptor expression						
PgR-positive	1.775 ± 0.480	0.890	44.93 ± 11.64	0.041*	0.038 ± 0.012	0.048*
PgR-negative	1.760 ± 0.633		43.73 ± 8.59		0.041 ± 0.016	
HER2 amplification						
HER2-positive	1.797 ± 0.643	0.364	43.66 ± 10.16	0.313	0.039 ± 0.017	0.100
HER2-negative	1.766 ± 0.489		44.93 ± 11.09		0.038 ± 0.012	
Ki67% expression						
Ki67 < 20%	1.757 ± 0.577	0.968	47.17 ± 1.02	0.240	0.038 ± 0.014	0.328
Ki67 ≥ 20%	1.800 ± 0.518		43.93 ± 10.06		0.038 ± 0.013	
TNM stage						
Stage I	1.652 ± 0.555	0.668	47.30 ± 12.75	0.035*	0.037 ± 0.011	0.143
Stage II	1.807 ± 0.489		45.47 ± 10.18		0.038 ± 0.014	
Stage III	1.697 ± 0.631		40.60 ± 9.90		0.042 ± 0.015	
T stage						
T1	1.800 ± 0.538	0.935	48.58 ± 12.36	0.024*	0.037 ± 0.011	0.352
T2	1.757 ± 0.516		44.47 ± 8.28		0.038 ± 0.014	
T3	1.677 ± 0.642		40.60 ± 12.31		0.039 ± 0.016	
T4	1.807 ± 0.550		41.23 ± 11.45		0.043 ± 0.015	
N stage						
N0	1.677 ± 0.515	0.736	44.67 ± 11.91	0.492	0.037 ± 0.013	0.538
N1	1.813 ± 0.503		44.90 ± 9.95		0.039 ± 0.013	
N2	1.705 ± 0.641		44.20 ± 7.63		0.044 ± 0.012	
N3	1.651 ± 1.106		39.5 ± 8.60		0.044 ± 0.022	
Scarf–Bloom–Richardson grade						
G1	1.520 ± 0.522	0.143	40.60 ± 8.33	0.843	0.034 ± 0.005	0.026*
G2	1.670 ± 0.605		44.40 ± 11.61		0.036 ± 0.015	
G3	1.840 ± 0.450		44.57 ± 10.20		0.038 ± 0.011	
Lymphovascular invasion						
Yes	1.820 ± 0.657	0.286	45.47 ± 8.73	0.694	0.039 ± 0.014	0.272
No	1.728 ± 0.500		44.35 ± 11.33		0.038 ± 0.013	
Pathological complete response to NAC						
Yes	1.813 ± 0.470	0.039*	44.43 ± 11.72	0.292	0.039 ± 0.012	0.367
No	1.650 ± 0.589		42.00 ± 8.90		0.038 ± 0.016	
Relapse						
Yes	1.783 ± 0.627	0.772	45.02 ± 6.46	0.987	0.039 ± 0.014	0.889
No	1.771 ± 0.524		44.35 ± 11.39		0.038 ± 0.014	

The corresponding p values for patients showing or not a pCR and for patients with or without relapse are reported in the table. A significance level of 0.05 is accepted (in italics)

Median value of Kyn, Trp and Kyn/Trp ratio according to patient and tumor characteristics, to response to chemotherapy and relapse. The p value was calculated as the mean of the Mann–Whitney U test for two variables or the Kruskal–Wallis test for three or more variables

*Kyn* kynurenine, *Trp* tryptophan, *SD* standard deviation, *TNBC* triple-negative breast cancer, *ER* estrogen receptor, *NAC* neoadjuvant chemotherapy

The Kyn and Kyn/Trp ratio in plasma showed a different distribution according to age in the entire cohort, with a lower plasmatic concentration of Kyn (1.738 ± 0.609 μM/L vs. 1.980 ± 0.643 μM/L, p < 0.0001) and Kyn/Trp ratio (0.036 ± 0.015 vs. 0.043 ± 0.016, p < 0.0001) for patients younger than 54.5 years (Table 2). Consistently, we observed a significant positive correlation between the age and circulating Kyn or Kyn/Trp ratio, with correlation coefficients of 0.295 (p < 0.0001) and 0.300 (p < 0.0001), respectively (Table 3 and Additional file 1: Figure S3).

**Table 3:** Correlation of Kyn, Trp and Kyn/Trp ratio with the continuous variables age, ER, PgR, Ki67 and size

	Kyn			Trp			Ratio		
	Correlation coefficient	95% CI	p value	Correlation coefficient	95% CI	p value	Correlation coefficient	95% CI	p value
Age (years)	*0.295*	*0.196 to 0.389*	*<* 0.0001***	0.028	− 0.078 to 0.133‏	0.610	*0.300*	*0.200 to 0.393*	*<* 0.0001***
ER (%)	0.010	− 0.129 to 0.148	0.890	*0.146*	*0.008 to 0.279*	0.039*	− 0.103	− 0.239 to 0.036	0.145
PgR (%)	− 0.074	− 0.211 to 0.065	0.297	0.116	− 0.023 to 0.250	0.103	*−* *0.184*	*−* *0.315 to −* *0.046*	0.009**
Ki67 (%)	− 0.036	− 0.173 to 0.103	0.614	− 0.108	− 0.243 to 0.031	0.128	0.045	− 0.94 to 0.183	0.523
Size (mm)	0.003	− 0.137 to 0.142	0.968	*−* *0.170*	*−* *0.302 to −* *0.031*	0.017*	0.119	− 0.021 to 0.254	0.096

The correlation was calculated with the Spearman test. The correlation coefficient, 95% CI and p value are reported in the table. The significant correlations are reported in italics

*CI* confidence interval, *ER* estrogen receptor, *PgR* progesterone receptor

In the cohort of cancerous patients, a statistically significant lower Trp level and higher Kyn/Trp ratio in plasma were observed for the ER− and PgR− patients compared to ER+ and PgR+ patients, respectively (Table 2). In particular, the values of circulating Trp were 42.00 ± 8.88 μM/L vs. 45.47 ± 11.26 μM/L (p 0.016) in ER− vs. ER+ and 43.73 ± 8.59 μM/L vs. 44.93 ± 11.64 μM/L (p 0.041) in PgR− and PgR+, respectively. The values of the plasmatic Kyn/Trp ratio were 0.042 ± 0.016 in ER− vs. 0.0377 ± 0.012 in ER+ patients (p 0.040) and 0.041 ± 0.016 in PgR− vs. 0.038 ± 0.012 in PgR+ patients (p 0.048). The correlation analysis showed a positive correlation between ER% and circulating Trp level and a negative correlation between PgR% and the Kyn/Trp ratio in plasma, as reported in Table 3 and in Additional file 1: Figure S3.

A lower plasmatic Kyn/Trp ratio for lobular carcinomas compared to the other histologies was also detected, particularly with a ratio of 0.033 ± 0.009 for lobular, 0.038 ± 0.014 for ductal and 0.038 ± 0.008 for other histologies (p 0.005). A comparison by pairs confirmed the difference between ductal and lobular carcinoma with a p value of 0.011 (Additional file 1: Table S1).

Moreover, the plasmatic Kyn/Trp ratio showed progressively higher values with increasing tumor grade, particularly 0.034 ± 0.005 for G1, 0.036 ± 0.015 for G2 and 0.038 ± 0.011 for G3 (p 0.026). The comparison by pairs did not confirm this difference (Additional file 1: Table S6), probably due to the low number of patients classified as G1.

We also observed a progressively lower circulating Trp level according to the increase of T (from 48.58 ± 12.36 μM/L in T1 to 41.23 ± 11.45 μM/L in T4, p 0.024) and TNM stage (from 47.30 ± 12.75 μM/L for Stage I to 40.60 ± 9.90 μM/L for Stage III, p 0.035). In particular, plasmatic Trp showed a significantly different distribution in the T1 tumor compared to T2-4 (p 0.003) and in Stage I compared to Stage II–III (p 0.014), according to the Mann–Whitney U test. The comparison by pairs showed a statistically significant difference between T1 and T2 and between Stage I and Stage III (Additional file 1: Tables S3, S4). Likewise, a negative correlation between the plasmatic Trp level and tumor size expressed in mm was observed (Table 3 and Additional file 1: Figure S3).

For all the other variables (BC subtype, HER2 expression, ki67 expression, N stage and lympho-vascular invasion), we did not observe any differences in circulating Kyn, Trp and the Kyn/Trp ratio distribution, as shown in Table 2 and in Additional file 1: Tables S1–S6.

In addition to the association of plasmatic Kyn, Trp and the Kyn/Trp ratio with cancer characteristics, we also analyzed their predictive roles in the response to chemotherapy and cancer relapse (Table 2). We observed a statistically significant higher circulating Kyn level for patients experiencing a pathological complete response (pCR) after NAC in 126 patients, with a value of 1.813 ± 0.470 μM/L vs. 1.650 ± 0.589 μM/L (p 0.039), but no differences in plasmatic Trp or the Kyn/Trp ratio were detected. Considering the different breast cancer subtypes, we detected a higher plasmatic Kyn (1.887 ± 0.327 μM/L vs. 1.580 ± 0.463 μM/L, p 0.001) and Kyn/Trp ratio (0.045 ± 0.011 vs. 0.036 ± 0.011, p 0.029) for Luminal B patients who reached a pCR compared with patients with residual disease, but not significant association for the other subtypes (Table 4). No significant difference was detected in the four subtypes between patients experiencing a relapse and those not (Table 4). Interestingly, we observed a higher Kyn median value, a lower Trp and a higher Kyn/Trp ratio in plasma in TNBC with residual disease and in patients who relapsed, even if the significance level was not reached.

**Table 4:** Kyn, Trp and Kyn/Trp ratio in breast cancer subtypes

	Group (N of pts)	Kyn		Trp		Kyn/Trp ratio	
		Median ± SD	p value	Median ± SD	p value	Median ± SD	p value
Luminal A (60 pts)	PCR (0)	NA	NA	NA	NA	NA	NA
	No pCR (13)	1.710 ± 0.684	NA	36.70 ± 8.26	NA	0.042 ± 0.019	NA
	No relapse (53)	1.775 ± 0.582	0.982	48.17 ± 13.02	0.442	0.038 ± 0.014	0.542
	Relapse (7)	1.717 ± 0.501		44.67 ± 5.88		0.043 ± 0.009	
Luminal B (89 pts)	PCR (23)	1.887 ± 0.327	0.001**	44.77 ± 14.17	0.455	0.045 ± 0.011	0.029*
	No pCR (42)	1.580 ± 0.463		43.47 ± 8.51		0.036 ± 0.011	
	No relapse (79)	1.807 ± 0.463	0.194	43.87 ± 10.53	0.447	0.038 ± 0.011	0.119
	Relapse (10)	1.653 ± 0.389		46.93 ± 8.42		0.035 ± 0.006	
HER2-enriched (14 pts)	PCR (7)	1.757 ± 0.790	0.234	39.73 ± 8.12	0.836	0.045 ± 0.018	0.295
	No pCR (6)	2.43 ± 0.956		42.42 ± 5.03		0.063 ± 0.028	
	No relapse (10)	1.828 ± 0.786	0.454	37.80 ± 6.67	0.076	0.047 ± 0.023	1
	Relapse (4)	2.398 ± 1.135		46.13 ± 2.94		0.049 ± 0.026	
TNBC (39 pts)	PCR (13)	1.707 ± 0.476	0.853	44.43 ± 7.98	0.511	0.037 ± 0.008	0.353
	No pCR (22)	1.777 ± 0.449		41.97 ± 10.87		0.042 ± 0.013	
	No relapse (39)	1.648 ± 0.450	0.707	44.78 ± 10.73	0.440	0.037 ± 0.012	0.761
	Relapse (7)	1.813 ± 0.437		42.00 ± 4.65		0.042 ± 0.011	

The corresponding p values for patients showing or not a pCR and for patients with or without relapse are reported in the table. A significance level of 0.05 is accepted (in italics)

Median value of Kyn, Trp and Kyn/Trp ratio in the different breast cancer subtypes according to the response to chemotherapy and relapse tested by the mean of the Mann–Whitney U test

*Kyn* kynurenine, *Trp* tryptophan, *SD* standard deviation, *PCR* pathological complete response, *TNBC* triple-negative breast cancer

**Fig. 2:**
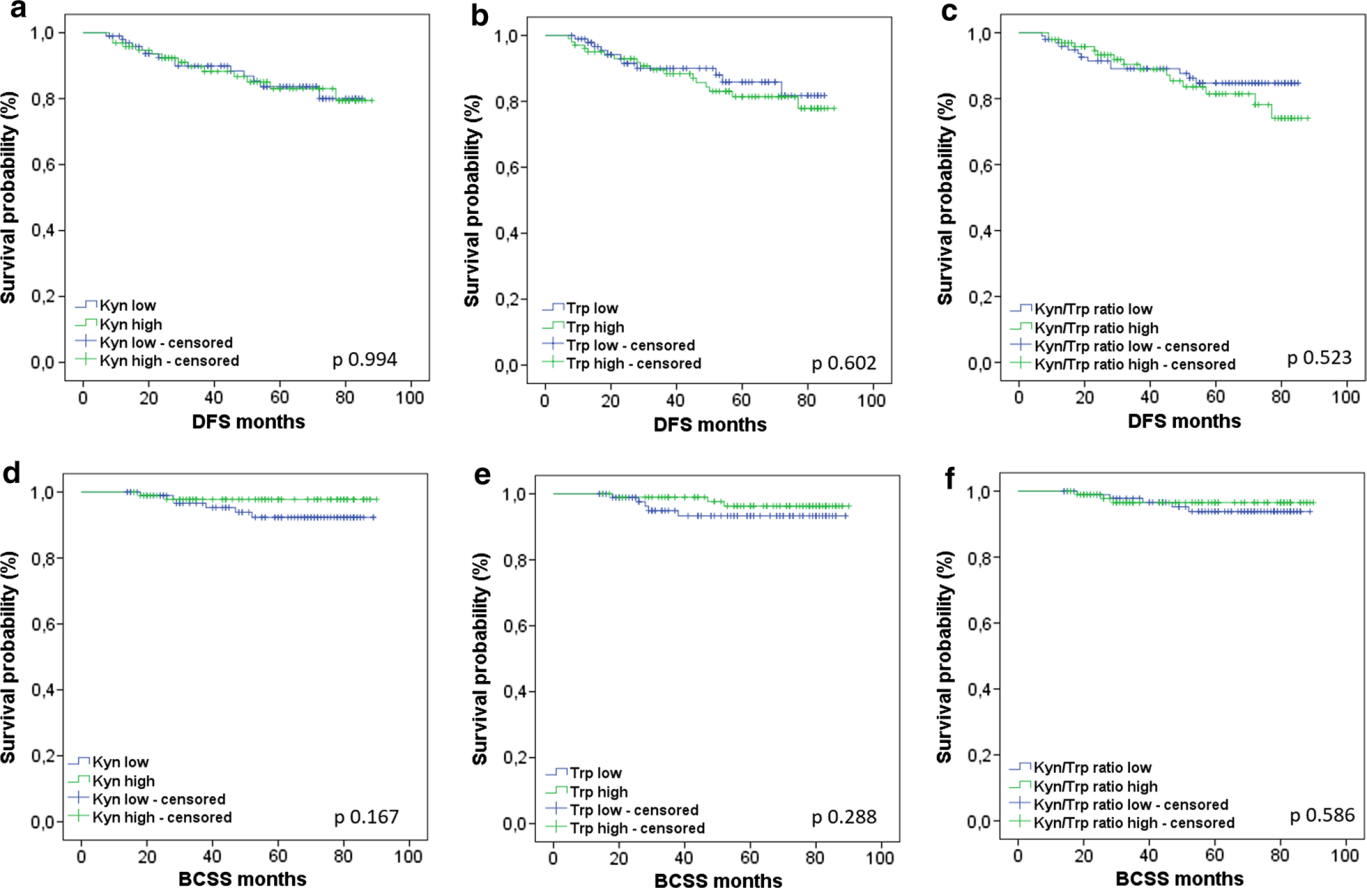
Kaplan–Meier curves for DFS according to Kyn (**a**), Trp (**b**) and the Kyn/Trp ratio (**c**) and for BCSS according to Kyn (**d**), Trp (**e**) and the Kyn/Trp ratio (**f**). The cohort was divided into Kyn low and high groups, Trp low and high groups and Kyn/Trp ratio low and high groups, using the median respective value for the cohort affected by breast cancer as a threshold. The statistical significance was calculated by means of a log-rank test for each curve, and the corresponding p values are reported in the figure

We classified BC patients into different groups according to circulating Kyn, Trp and the Kyn/Trp levels using the median value in the cancerous patients as the threshold: 1.77 μM/L for Kyn, 44.56 μM/L for Trp and 0.038 for the Kyn/Trp ratio. We analyzed DFS and BCSS in these groups. Six patients of 202 of the BC cohort were not evaluable for survival due to deaths from other causes or loss during follow-up. Among the 196 patients included in the analysis for survival, we detected 28 progressions and 8 deaths. We detected a non statistically significant difference in DFS in the groups with low and high plasmatic Kyn/Trp ratio (Fig. 2c), with a 5-year DFS of 85% vs. 73% (p 0.523), respectively. For all the other variables (low and high circulating kyn and Trp), we did not observe any difference in the groups analyzed (Fig. 2 and Table 5).

**Table 5:** DFS and BCSS according to Kyn, Trp and the Kyn/Trp ratio

Outcome	Parameter	Kyn			Trp			Kyn/Trp ratio		
		Low	High	p value	Low	High	p value	Low	High	p value
DFS	N patients	100	96	NA	95	101	NA	98	98	NA
	N events	14	14	NA	11	17	NA	13	15	NA
	5-year-DFS	80%	79%	0.994	81%	78%	0.602	85%	73%	0.523
	HR (95% CI)	1.003 (0.477–2.106)			1.224 (0.572–2.616)			1.273 (0.605–2.679)		
BCSS	N patients	94	94	NA	90	98	NA	93	95	NA
	N events	6	2	NA	5	3	NA	5	3	NA
	5-year-BCSS	92%	98%	0.167	94%	96%	0.288	94%	97%	0.586
	HR (95% CI)	0.341 (0.069–1.691)			0.469 (0.112–1.964)			0.673 (0.161–2.822)		

*Kyn* kynurenine, *Trp* tryptophan, *DFS* disease-free survival, *N* number, *HR* hazard ratio, *BCSS* breast cancer-specific survival

The analysis of DFS and BCSS in the different BC subtypes according to circulating Kyn, Trp and the Kyn/Trp ratio did not show significant results (Additional file 1: Figures S1, S2).

## Discussion

In this study, we analyzed Kyn and Trp levels and their ratios in the plasma of early BC patients and healthy CTRLs. To our knowledge, this is the largest study evaluating the plasmatic level of Trp and its metabolite Kyn in this setting of patients. The aim of our study was to identify a difference in circulating Kyn, Trp and the Kyn/Trp level in the presence of a BC, according to BC characteristics and their predictive role on survival, as observed in other malignancies.

We met our primary objective to demonstrate the difference in plasmatic Kyn, Trp and the Kyn/Trp ratio between BC patients and healthy CTRLs. Overall, we observed lower Kyn and Trp levels in plasma of cancerous patients, suggesting rapid Trp catabolism, with a possible accumulation of downstream metabolites, such as quinolinic and kynurenic acid. The same results were shown by Greene and colleagues in a cohort of 77 BC patients compared to 40 healthy CTRLs [24]. These authors also analyzed the plasma level of nicotinamide, the final metabolite of Trp catabolism. They observed an accumulation of nicotinamide relative to the Trp level in plasma of BC patients, which suggests hyperactivation of the catabolic pathway [24]. In our study, we also found a higher plasmatic Kyn/Trp ratio in healthy CTRLs than in BC patients. These results are not consistent with those shown in a previously published study by Lyon and colleagues, but we note that the sample in this paper comprised only 57 subjects vs. 348 in our study [23]. Our finding of a lower Kyn/Trp ratio in BC concomitant to lower Kyn and Trp levels in plasma suggests a major decrease in Kyn vs. Trp in the presence of cancer, probably due to the hyperactivation of other downstream enzymes, such as kynureninase (KYNU) and kynurenine hydroxylase. This is consistent with the results of D’Amato and colleagues, who observed an upregulation not only of TDO but also of KYNU in TNBC cell lines [26].

In our study, we observed a higher plasmatic Kyn and Kyn/Trp ratio in older women. This result is consistent with literature data showing an increase in tryptophan metabolism with age, which is known to be associated with central nervous system degenerative diseases [27]. Analogously, an accumulation of Kyn and other Trp products in serum was also observed in aging mice [28].

Hormone receptor (HR) status is also related to Trp catabolism, as already shown in the literature, with an upregulation of TDO in an NF-κB-dependent manner in HR- breast cancer cell lines [26]. This is consistent with our finding of a lower circulating Trp level concomitant with a higher Kyn/Trp ratio in HR− tumors. Considering the known immunosuppressive role of Kyn and the fact that HR− BCs are associated with a higher immune infiltration, we could not associate the difference in immune system activation throughout breast cancer subtypes with the plasmatic Kyn/Trp ratio. In fact, plasmatic Kyn and Trp levels reflect the systemic Trp catabolism and could not be considered a reflection of tumor microenvironment, in which the local enzymatic activity determines the level of metabolites at tumor site.

In stage I tumors compared to Stage II and III tumors and in T1 tumors compared to T2-4 tumors, we observed a higher plasmatic Trp level, suggesting faster Trp degradation in larger tumors.

In the whole cohort of patients receiving NAC, we observed a higher level of circulating Kyn in patients experiencing a pCR without any statistically significant difference in Trp level or in the Kyn/Trp ratio in plasma, while in the Luminal B subtype only, the high Kyn level for patients with pCR was concomitant with a significant increase in the Kyn/Trp ratio in plasma samples. This is in contrast with what was expected, considering the immunosuppressive role of Kyn and the known association of pCR with TIL infiltration [29, 30]. In addition, in TNBC with residual disease and with relapse, it is interesting to observe a higher Kyn median value, a lower Trp and a higher Kyn/Trp ratio. However, the TNBC subgroup of our cohort comprised only 39 patients, and these results did not reach significance. Nevertheless, this study analyzes only Trp and its metabolites in plasma, and we cannot exclude a role for Trp catabolism in the tumor microenvironment.

Finally, we did not observe any association between plasmatic Kyn, Trp or their ratio with survival, in contrast to what was observed for other cancer types. In fact, previously published studies showed a significant association between a low Kyn/Trp ratio in plasma and longer survival in non-small cell lung cancer (NSCLC) treated with chemoradiation [14, 15] or immunotherapy [16].

The present study is the largest analyzing plasmatic Kyn and Trp levels in BC and in healthy CTRLs. This high number of patients allows us to compare the plasmatic concentrations of these metabolites between breast cancer subtypes, disease stage, proliferation marker expression, tumor grade, clinical outcome and response to chemotherapy. In no other previous studies the Trp and Kyn levels were so well described according to tumor characteristics, probably due to the smallest sample size. The plasmatic Trp level could be related to fasting, that is a missing information in our cohort. In the second instance, we did not measure the circulating downstream metabolites after Kyn, which could be useful for better understanding the variations in Trp catabolism at any level of the pathway. Moreover, we have to take into account that other enzymes are involved in Trp catabolism, as well the metabolic pathway at the tumor level could be different from systemic catabolism and not necessarily reflected in circulating metabolite-level modification. Consequently, we cannot consider plasmatic Kyn and Trp levels as a direct reflection of IDO activity in tumor tissue. An analysis of the tumor site in addition to the plasmatic measurement of Trp and its metabolites could be useful to clarify the correspondence between the circulating and tumor compartments. Finally, a longer follow-up could be useful for the survival analysis. In our cohort, we observed only 28 events of progression and 8 of death out of 196 patients evaluated for outcome, which did not allow us to obtain conclusive results regarding survival.

## Conclusions

In our study, we showed that plasmatic levels of Kyn, Trp and the Kyn/Trp ratio could differentiate BC patients from healthy CTRLs, but they were not useful for predicting the outcome or sensitivity to chemotherapy. According to the above observations, breast cancer is associated with rapid metabolism that could be systemic or local.

## Additional file

**Additional file 1: Table S1.** Comparison by pairs of Kyn, Trp and Kyn/Trp distributions according to histology. **Table S2.** Comparison by pairs of Kyn, Trp and Kyn/Trp distributions according to breast cancer subtype. **Table S3.** Comparison by pairs of Kyn, Trp and Kyn/Trp distributions according to tumor stage. **Table S4.** Comparison by pairs of Kyn, Trp and Kyn/Trp distributions according to T stage. **Table S5.** Comparison by pairs of Kyn, Trp and Kyn/Trp distributions according to N stage. **Table S6.** Comparison by pairs of Kyn, Trp and Kyn/Trp distributions according to Scarf–Bloom–Richardson tumor grade. **Figure S1.** Forest plot for DFS according to the 4 breast cancer subtypes and for Kyn, Trp and the Kyn/Trp ratio. **Figure S2.** Forest plot for BCSS according to the 4 breast cancer subtypes and for Kyn, Trp and the Kyn/Trp ratio. **Figure S3.** Correlation graphs for Kyn, Trp and the Kyn/Trp ratio with age, ER, PgR, Ki67 and size.

## Data Availability

The datasets used and/or analyzed during the current study are available from the corresponding author upon reasonable request.

## Abbreviations

AHR: aryl hydrocarbon receptor
BCSS: breast cancer specific survival
CTRL: control
DC: dendritic cell
DFS: disease free survival
ER: estrogen receptor
HR: hormone receptor
IDO: indoleamine 2,3-dioxygenase
ILT3: immunoglobulin-like transcript 3
ILT4: immunoglobulin-like transcript 4
Kyn: kynurenine
KYNU: kynureninase
NAC: neoadjuvant chemotherapy
PCR (or pCR): pathological complete response
PgR: progesterone receptor
TDO: tryptophan 2,3-dioxygenase
TNBC: triple negative breast cancer
Treg: T regulatory
Trp: tryptophan
