# Supplementary material for: Tryptophan catabolism increases in breast cancer patients compared to healthy controls without affecting the cancer outcome or response to chemotherapy
Source: J Transl Med. 2019 Jul 23;17:239. doi: 10.1186/s12967-019-1984-2 (PMC6652004; doi:10.1186/s12967-019-1984-2)
Supplement: Supplementary file 1 — Additional file 1: Table S1. Comparison by pairs of Kyn, Trp and Kyn/Trp distributions according to histology. Table S2. Comparison by pairs of Kyn, Trp and Kyn/Trp distributions according to breast cancer subtype. Table S3. Comparison by pairs of Kyn, Trp and Kyn/Trp distributions according to tumor stage. Table S4. Comparison by pairs of Kyn, Trp and Kyn/Trp distributions according to T stage. Table S5. Comparison by pairs of Kyn, Trp and Kyn/Trp distributions according to N stage. Table S6. Comparison by pairs of Kyn, Trp and Kyn/Trp distributions according to Scarf–Bloom–Richardson tumor grade. Figure S1. Forest plot for DFS according to the 4 breast cancer subtypes and for Kyn, Trp and the Kyn/Trp ratio. Figure S2. Forest plot for BCSS according to the 4 breast cancer subtypes and for Kyn, Trp and the Kyn/Trp ratio. Figure S3. Correlation graphs for Kyn, Trp and the Kyn/Trp ratio with age, ER, PgR, Ki67 and size. [file 12967_2019_1984_MOESM1_ESM.docx]

**Additional Materials**

[Table S1. Comparison by pairs of Kyn, Trp and Kyn/Trp distributions according to histology. 2](#_Toc12818792)

[Table S2. Comparison by pairs of Kyn, Trp and Kyn/Trp distributions according to breast cancer subtype. 2](#_Toc12818793)

[Table S3. Comparison by pairs of Kyn, Trp and Kyn/Trp distributions according to tumor stage. 2](#_Toc12818794)

[Table S4. Comparison by pairs of Kyn, Trp and Kyn/Trp distributions according to T stage. 3](#_Toc12818795)

[Table S5. Comparison by pairs of Kyn, Trp and Kyn/Trp distributions according to N stage. 3](#_Toc12818796)

[Table S6. Comparison by pairs of Kyn, Trp and Kyn/Trp distributions according to Scarf-Bloom-Richardson tumor grade. 3](#_Toc12818797)

[Figure S1. Forest plot for DFS according to the 4 breast cancer subtypes and for Kyn, Trp and the Kyn/Trp ratio. 4](#_Toc12818798)

[Figure S2. Forest plot for BCSS according to the 4 breast cancer subtypes and for Kyn, Trp and the Kyn/Trp ratio. 5](#_Toc12818799)

[Figure S3. Correlation graphs for Kyn, Trp and the Kyn/Trp ratio with age, ER, PgR, Ki67 and size. 6](#_Toc12818800)

# **Table S1. Comparison by pairs of Kyn, Trp and Kyn/Trp distributions according to histology.**

| **Comparison** | **P value for Kyn** | **P value for Trp** | **P value for Kyn/Trp ratio** |
| --- | --- | --- | --- |
| **Ductal vs Lobular** | 0.184 | 0.211 | **0.011*** |
| **Ductal vs other** | 0.518 | 0.728 | 1 |
| **Lobular vs other** | 1 | 0.146 | 0.751 |

Kyn: kynurenine; Trp: tryptophan

The Kyn, Trp and Kyn/Trp ratio distribution were tested in the different histologies with a comparison by pairs, performed through the Bonferroni test. The corresponding p values are reported in the table. The statistically significant values are reported in bold.

# **Table S2. Comparison by pairs of Kyn, Trp and Kyn/Trp distributions according to breast cancer subtype.**

| **Comparison** | **P value for Kyn** | **P value for Trp** | **P value for Kyn/Trp ratio** |
| --- | --- | --- | --- |
| **Luminal A vs Luminal B** | 1 | 1 | 1 |
| **Luminal A vs HER2-enriched** | 0.163 | 0.174 | **0.001**** |
| **Luminal A vs TNBC** | 0.866 | 0.302 | 1 |
| **Luminal B vs HER2-enriched** | **0.026*** | 0.708 | **< 0.0001***** |
| **Luminal B vs TNBC** | 1 | 1 | 1 |
| **Her2-enriched vs TNBC** | **0.014*** | 1 | **0.002**** |

Kyn: kynurenine; Trp: tryptophan; TNBC: Triple negative breast cancer

The Kyn, Trp and Kyn/Trp ratio distribution were tested in the different breast cancer subtypes with a comparison by pairs, performed through the Bonferroni test. The corresponding p values are reported in the table. The statistically significant values are reported in bold.

# **Table S3. Comparison by pairs of Kyn, Trp and Kyn/Trp distributions according to tumor stage.**

| **Comparison** | **P value for Kyn** | **P value for Trp** | **P value for Kyn/Trp ratio** |
| --- | --- | --- | --- |
| **Stage I vs stage II** | 1 | 0.571 | 0.477 |
| **Stage I vs stage III** | 1 | **0.024*** | 0.127 |
| **Stage II vs stage III** | 1 | 0.180 | 0.941 |

Kyn: kynurenine; Trp: tryptophan

The Kyn, Trp and Kyn/Trp ratio distribution were tested according to tumor stage with a comparison by pairs, performed through the Bonferroni test. The corresponding p values are reported in the table. The statistically significant values are reported in bold.

# **Table S4. Comparison by pairs of Kyn, Trp and Kyn/Trp distributions according to T stage.**

| **Comparison** | **P value for Kyn** | **P value for Trp** | **P value for Kyn/Trp ratio** |
| --- | --- | --- | --- |
| **T1 vs T2** | 1 | **0.022*** | 0.898 |
| **T1 vs T3** | 1 | 0.382 | 0.902 |
| **T1 vs T4** | 1 | 0.070 | 0.264 |
| **T2 vs T3** | 1 | 1 | 1 |
| **T2 vs T4** | 1 | 1 | 1 |
| **T3 vs T4** | 1 | 1 | 1 |

Kyn: kynurenine; Trp: tryptophan

The Kyn, Trp and Kyn/Trp ratio distribution were tested according to T stage with a comparison by pairs, performed through the Bonferroni test. The corresponding p values are reported in the table. The statistically significant values are reported in bold.

# **Table S5. Comparison by pairs of Kyn, Trp and Kyn/Trp distributions according to N stage.**

| **Comparison** | **P value for Kyn** | **P value for Trp** | **P value for Kyn/Trp ratio** |
| --- | --- | --- | --- |
| **N0 vs N1** | 1 | 1 | 1 |
| **N0 vs N2** | 1 | 1 | 1 |
| **N0 vs N3** | 1 | 0.811 | 1 |
| **N1 vs N2** | 1 | 1 | 1 |
| **N1 vs N3** | 1 | 0.880 | 1 |
| **N2 vs N3** | 1 | 1 | 1 |

Kyn: kynurenine; Trp: tryptophan

The Kyn, Trp and Kyn/Trp ratio distribution were tested according to N stage with a comparison by pairs, performed through the Bonferroni test. The corresponding p values are reported in the table. No statistically significant values were detected.

# **Table S6. Comparison by pairs of Kyn, Trp and Kyn/Trp distributions according to Scarf-Bloom-Richardson tumor grade.**

| **Comparison** | **P value for Kyn** | **P value for Trp** | **P value for Kyn/Trp ratio** |
| --- | --- | --- | --- |
| **G1 vs G2** | 1 | 1 | 1 |
| **G1 vs G3** | 0.790 | 1 | 0.648 |
| **G2 vs G3** | 1 | 0.806 | 0.508 |

Kyn: kynurenine; Trp: tryptophan

The Kyn, Trp and Kyn/Trp ratio distribution were tested according to tumor grade with a comparison by pairs, performed through the Bonferroni test. The corresponding p values are reported in the table. No statistically significant values were detected.

#
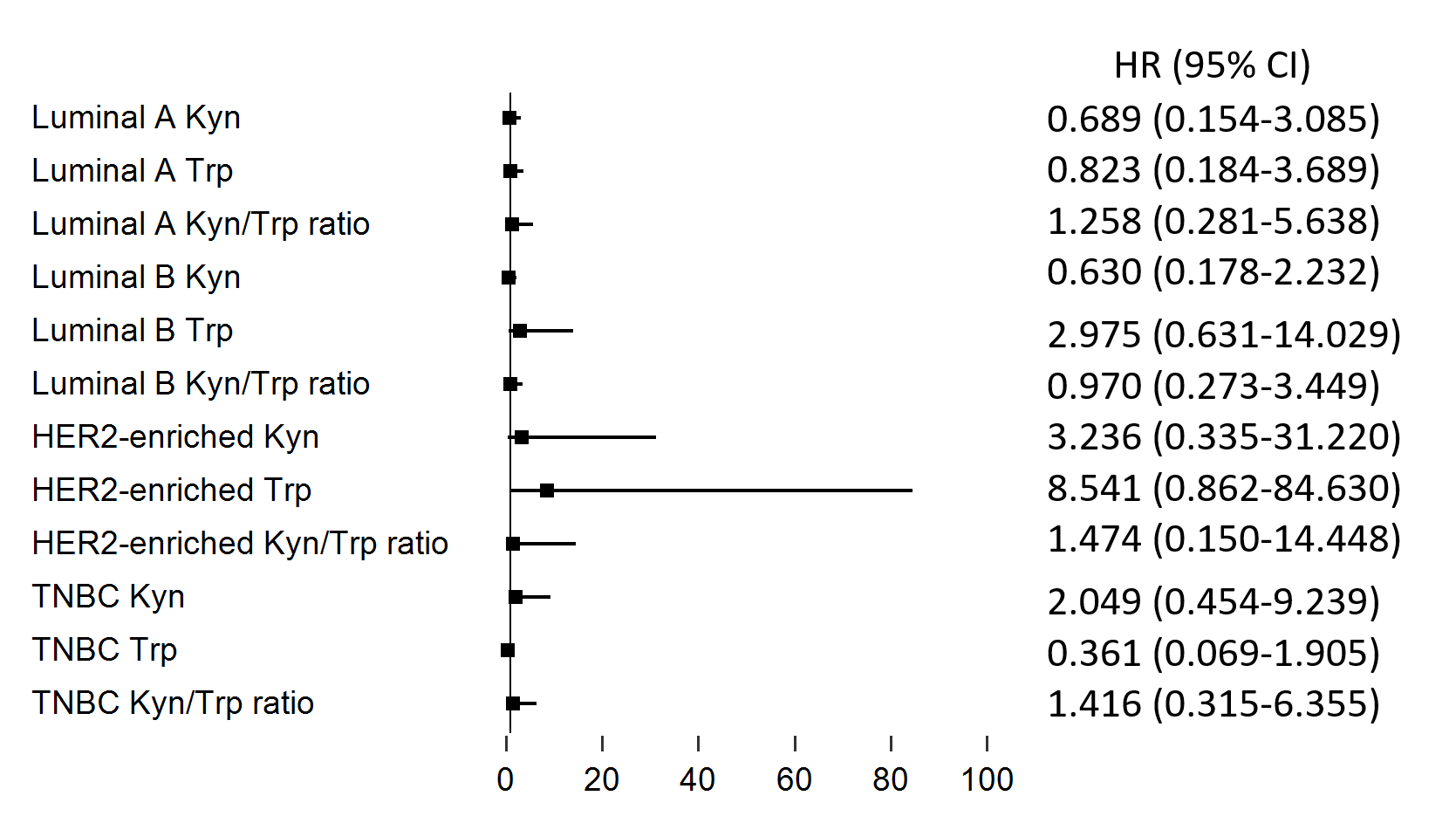
**Figure S1. Forest plot for DFS according to the 4 breast cancer subtypes and for Kyn, Trp and the Kyn/Trp ratio.**

The HR and 95% confidence interval for DFS are represented in the forest plot according to the breast cancer subtype and Kyn, Trp and Kyn/Trp ratio. The corresponding values are reported on the right side of the figure. An HR < 1 and > 0 indicates a benefit for the level of Kyn, Trp or Kyn/Trp ratio higher than the median value, while a value of HR > 1 corresponds to a benefit for the lower level of Kyn, Trp and their ratio. The vertical line represents the cut-off for an HR of 1.

# **Figure S2. Forest plot for BCSS according to the 4 breast cancer subtypes and for Kyn, Trp and the Kyn/Trp ratio.**


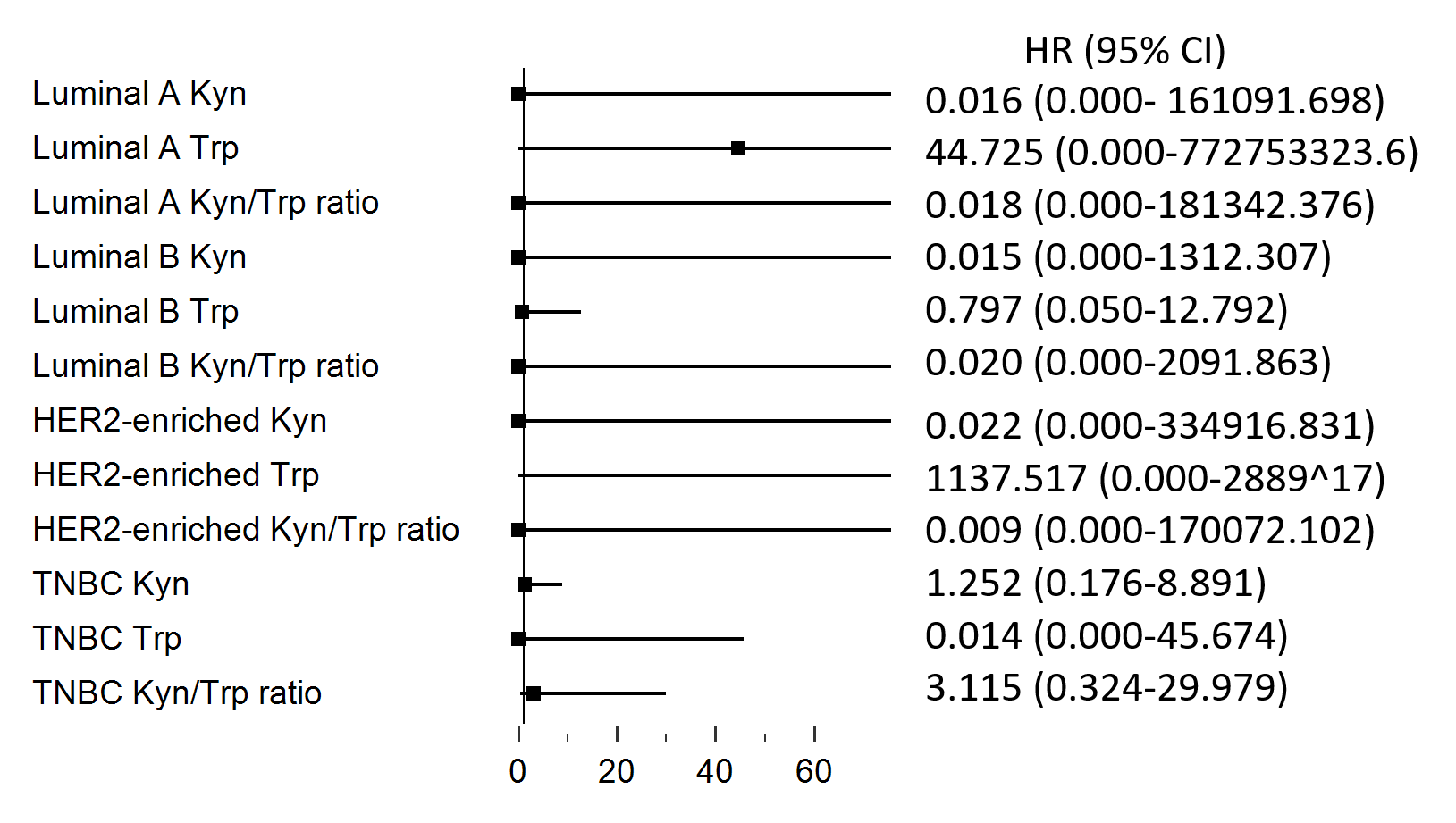


The HR and 95% confidence interval for BCSS are represented in the Forest plot according to breast cancer subtype and Kyn, Trp and Kyn/Trp ratio. The corresponding values are reported on the right side of the figure. An HR < 1 and > 0 indicates a benefit for the level of Kyn, Trp or Kyn/Trp ratio higher than the median value, while a value of HR > 1 corresponds to a benefit for the lower level of Kyn, Trp and their ratio. The vertical line represents the cut-off for an HR of 1. The results are zoomed for a 95% CI value < 80.

# **Figure S3. Correlation graphs for Kyn, Trp and the Kyn/Trp ratio with age, ER, PgR, Ki67 and size.**


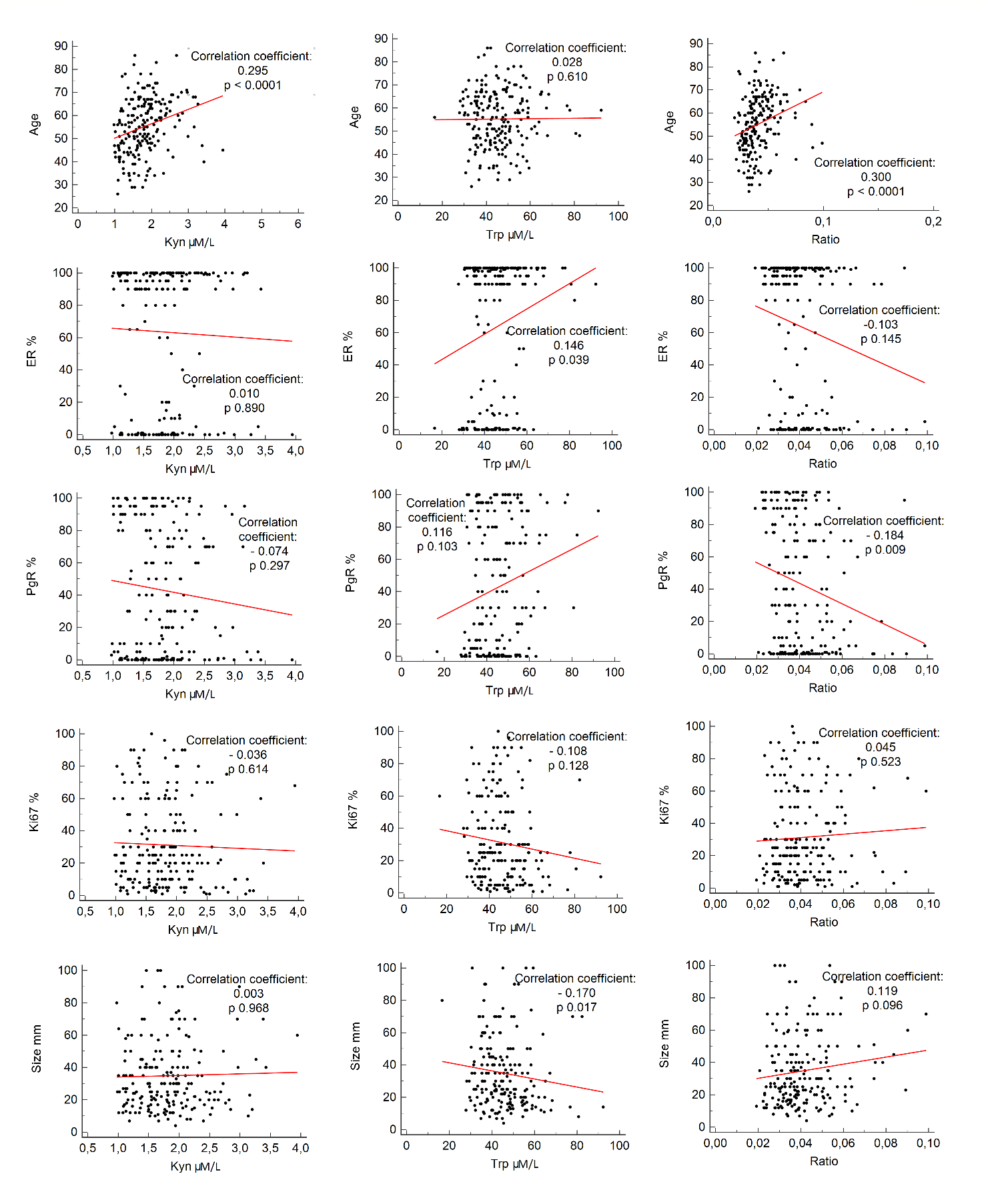


The correlation was calculated with the Spearman test. The corresponding correlation coefficient and p value are reported in each figure.
